# Do Hypertension, diabetes mellitus and obesity increase the risk of severity of nephrolithiasis?

**DOI:** 10.12669/pjms.313.7086

**Published:** 2015

**Authors:** Eyup Burak Sancak, Mustafa Reşorlu, Alpaslan Akbas, Murat Tolga Gulpinar, Muhammet Arslan, Berkan Resorlu

**Affiliations:** 1 Eyup Burak Sancak, Department of Radiology, Canakkale Onsekiz Mart University, Faculty of Medicine, Terzioglu Yerleskesi, Barbaros Mh, 17100, Canakkale, Turkey; 2 Mustafa Reşorlu, Department of Radiology, Canakkale Onsekiz Mart University, Faculty of Medicine, Terzioglu Yerleskesi, Barbaros Mh, 17100, Canakkale, Turkey; 3 Alpaslan Akbas, Department of Urology, Canakkale Onsekiz Mart University, Faculty of Medicine, Terzioglu Yerleskesi, Barbaros Mh, 17100, Canakkale, Turkey; 4 Murat Tolga Gulpinar, Department of Urology, Canakkale Onsekiz Mart University, Faculty of Medicine, Terzioglu Yerleskesi, Barbaros Mh, 17100, Canakkale, Turkey; 5 Muhammet Arslan, Vefa Hospital, Soma, Turkey; 6 Berkan Resorlu, Department of Urology, Canakkale Onsekiz Mart University, Faculty of Medicine, Terzioglu Yerleskesi, Barbaros Mh, 17100, Canakkale, Turkey

**Keywords:** Body mass index, Diabetes mellitus, Hypertension, Kidney stone, Stone burden

## Abstract

**Objective:**

In this study we planned to investigate the relationship between presence of kidney stones and stone burden with hypertension (HT), diabetes mellitus (DM) and body mass index (BMI).

**Methods:**

A total of 574 patients were included in the study. None of the patients had a history of stones. The 121 patients with kidney stone identified on ultrasound evaluation and the 453 patients with no stones were compared in terms of HT, BMI and DM. The stone burden of 121 patients with diagnosed stones was compared in terms of the same variables.

**Results:**

Of the 121 patients with kidney stones 30 (24.7%) had HT, while 66 (14.5%) of the 453 patients without stones had HT (p=0.007). BMI values of those with and without stones were 27.2 ± 4.93 kg/m^2^ and 25.29 ± 4.12 kg/m^2^, respectively (p<0.001). Twenty-five (20.6%) of the patients with stones diagnosed by ultrasound had DM, while 49 (10.8%) of those without stones had DM (p=0.004). When comparing patients with and without kidney stones, logistic regression analysis revealed that DM (odds ratio [OR] 2.06, 95% confidence interval [CI] 1.17 to 3.63, p=0.013) and BMI (OR 1.08, CI 1.03 to 1.13, p=0.003) were independently associated with presence of stones. No significant relationship was found between the same variables and cumulative stone diameter (CSD) and stone surface area (SA) evaluated for stone burden.

**Conclusions:**

While diabetes mellitus, Hypertension and increased Body Mass Index may add to the possibility of stone formation, they did not affect stone burden.

## INTRODUCTION

Kidney stones are a common disease with increasing incidence throughout the world and may cause significant health care burden especially in the working-age population. In the last 30 years the prevalence of stones in the USA has doubled and the lifetime formation risk of kidney stones is 6-12%.^1^-^5^ In recent years the responsibility for this increase in the incidence and prevalence of kidney stone disease has been blamed on dietary habits changes in lifestyle and insulin resistance.^4^,^6^-^9^

Metabolic syndrome (MS) affects 25% of the American population and is a common systemic disease that has been the topic of much scientific research in recent years. The relationship between components of MS such as obesity, Type 2 diabetes mellitus (DM) and hypertension (HT) and kidney stone disease has been shown in previous studies. However the relationship between the presence of these diseases and stone burden has not been investigated. A variety of parameters such as cumulative stone diameter (CSD), stone surface area (SA) and stone volume are used as markers for stone burden.^10^,^11^

In this study we planned to investigate the relationship between the presence of kidney stones and stone burden with HT, DM and body mass index.

## METHODS

### Study Population

Between December 2013 and April 2014, 630 patients above the age of 18 years who visited to the urology clinics of two reference hospitals and had renal US completed due to complaint of flank pain were retrospectively scanned. Patients with risk factors for kidney stones such as ureteropelvic junction obstruction, polycystic renal disease, those with renal malformations such as horseshoe kidney, malrotated or ectopic kidney or with glomerular or tubular renal disease and systemic diseases such as gout, neoplasms, malabsorption, thyroid or parathyroid disorders and sarcoidosis were not included in the study. Twenty-nine patients with no kidney stone found on ultrasound evaluation but with history of stones were excluded from the study. Sixteen patients with stone found and history of stones whose stone size may affect the evaluation were excluded from the study. Eleven patients were excluded due to incomplete data.

Finally the 574 patients included in the evaluation were divided into two groups depending on the presence or absence of stones on the basis of imaging results from USG and radiography of the kidneys, ureters and bladder (KUB). According to routine practice in the clinics, patients with suspected urolithiasis on ultrasound were sent for non-contrast computed tomography imaging. While 121 patient s (21.1%) had stones diagnosed, 453 (78.9%) patients did not have any stone found in the urinary system. For patients with stones, stone burden was evaluated based on cumulative stone diameter (CSD) and stone surface area (SA) parameters based on USG and KUB images. CSD was calculated by measuring the longest side of stones (in mm) for patients with a single stone and by adding together the sizes of longest stone side in patients with more than one stone. SA was calculated from the formula stone diameter x width x Ω x 1/4 (in mm^2^). For patients with several stones the SA values were added together.

The patients’ age, occupation, body mass index (BMI), chronic diseases such as hypertension (HT) and type 2 diabetes mellitus (DM) were recorded in the patients’ files. Age was recorded as that at last birthday. Occupation was categorized as student, retired/unemployed, officer, worker and tradesman. BMI was calculated as weight (kg) divided by the square of height in meters (kg/m^2^). Any measurement of systolic blood pressure ≥ 130 mmHg or diastolic blood pressure ≥ 85 mmHg or both or those taking regular medication treatment for HT were included in the HT patient group. Those with fasting blood glucose above 126 mg/dl and/or using medication for DM were evaluated as diabetic patients. Additionally BMI <18.5 kg/m^2^ was considered underweight, 18.5–24.9 kg/m^2^ was normal, 25.0 – 29.9 kg/m^2^ was overweight, 30.0 – 39.9 kg/m^2^ was obese and >40 kg/m^2^ was morbidly obese. All these variables were evaluated to determine whether they were risk factors for the presence of stones in the urinary system. Additionally these variables were evaluated in terms of stone burden in patients with stones.

### Ultrasound measurements

All examinations were performed by two radiologists with at least ten years experience in the field of ultrasound. Sonographic examinations were performed with grey scale ultrasound machines (Toshiba Aplio XG and General Electric Logiq 9) using two convex transducers at frequencies of 3.5 MHz and 4.0 MHz. The presence of stone was defined as an echogenic image with or without posterior acoustic shadowing, clearly located within the urinary tract.

### Statistical analysis

All statistical analyses were performed using SPSS, version 20.0. All values are shown as mean ± standard deviation or in situations in which the distributions were skewed, as the median (min - max). The Kolmogorov-Smirnov test was used to determine whether the distribution of our patient samples were normal or not. The significance of the differences between the groups with and without stones for hypertension, occupation, gender and cathegorical BMI were determined by chi-square test (or Fisher’s exact test; when chi-square test assumptions do not hold due to low expected cell counts). For the multivariate analysis, the possible factors identified with univariate analyses were further entered into the logistic regression analysis to determine independent predictors of patient outcome. Hosmer-Lemeshow goodness of fit statistics were used to assess model fit. For two-way group comparisons to determine the relationship between presence of stone with BMI and age the Mann-Whitney U analysis was completed. As the CSD and SA measurements were not normally distributed the Kruskal-Wallis tests were conducted to compare these parameters and the ordinal variables among the occupation status (6 subgrup) and categorical BMI (5 subgrup) groups. The Mann-Whitney U test was performed to test the significance of pair wise differences using Bonferroni correction to adjust for multiple comparisons. Comparisons of CSD and SA with BMI were completed with the Spearman correlation analysis. Lowest statistical significance level was accepted as p <0.05.

## RESULTS

Our study comprised a total of 574 patients with 306 male (53.3%) and 268 female (46.7%). The mean age was 50.34±16.60 (18-88 years). Of patients 75 (13.0%) had DM while 96 (16.7%) had HT. The average BMI of patients was calculated as 25.74±4.46. While urinary system US evaluation of all patients identified 121 (21.1%) with stones, no stone was found in the urinary system of 453 (78.9%) patients. The average CSD of patients with stones was 9.31±6.42 mm, while the average SA was calculated as 62.02±109.19 mm^2^. The mean age of patients with stones was 52.69 ± 17.96 years, while those without stones had a mean age of 49.71±16.18 years (p=0.109). There was a slight positive correlation between presence of stone and gender (p=0.031). There was no statistically significant relationship found between presence of stone and occupation (p=0.063).30 of the 121 (24.7%) patients with kidney stones had HT, while 66 (14.5%) of the 453 patients without stones had HT (p=0.007). However there was no significant relationship found between CSD and SA with HT (p=0.443, p=0.344, respectively). Twenty-five (20.6%) of the patients with stones diagnosed on US had DM while 49 (10.8%) of those without stones had DM (p=0.004). There was no significant relationship found between DM with CSD and SA (p=0.062, p=0.084). BMI values of those with and without stones were 27.2 ± 4.93 kg/m^2^ and 25.29±4.12 kg/m^2^, respectively (p<0.001). There was no significant relationship found between CSD and SA with BMI (p=0.400, p=0.141, respectively). The demographic data of the patients are given in Table-I.

**Table-I:** Patient demographics.

Variable	Kidney Stone		P value
	Yes	No	
No. patients	121 (21.1%)	453 (78.9%)	
Mean age ± SD (years)	52.69 ± 17.96	49.71 ± 16.18	0.109
Gender			0.031
• Man	75 (24.5%)	306	
• Women	46 (17.1%)	268	
Occupation (%)			
• Student	4 (8.3%)	44	0.063
• Housewife	38 (19.4%)	158	
• Retired / Unemployed	43 (25.3%)	127	
• Officer	12 (20.3%)	47	
• Worker	18(21.2%)	67	
• Tradesman	6(37.5%)	10	
Body mass index (mean)	27.2kg/m^2^	25.2 kg/m^2^	<0.001
• Underweight (<18.5)	1 (7%)	14	
• Normal weight (18.5 – 24.9)	35 (17%)	173	
• Overweight (25 – 29.9)	49 (19%)	206	
• Obesity (30 – 34.9)	32 (36%)	58	
• Severely obese (≥ 35)	4 (67%)	2	
Diabetes mellitus (%)	25 (34%)	74	0.004
Hypertension (%)	30 (35%)	66	0.007

* Statistically significant at p < 0.05.

According to logistic regression analysis to evaluate the independent predictors for the presence of stones, diabetes and BMI was found to increase the risk of kidney stone formation (respectively odds ratio [OR] 2.06, 95% confidence interval [CI] 1.17 to 3.63, p=0.013 and OR 1.08, CI 1.03 to 1.13, p=0.003) (Table II). There was a significant difference between patients with and without stones in terms of categorical BMI (p<0.001). Cumulative stone diameter (CSD) and stone surface area (SA) results were given in Table III.

**Table-II:** Logistic regression results in terms of the presence of stones.

Risk Factors	OR (95% CI)*	P value**
Age	0.99 (0.99-1.01)	0.532
Gender (man vs. women)	1.87 (0.89-3.93)	0.100
Body mass index	1.08 (1.03-1.13)	0.003
Diabetes mellitus	2.06 (1.17-3.63)	0.013
Hypertension	1.67 (0.98-2.84)	0.059

* Odds ratio, 95% Confident Interval

** Statistically significant at p < 0.05

**Table-III:** Cumulative stone diameter (CSD) and stone surface area (SA) results.

Variable	n	CSD*	P value	SA**	P value**
No. patients	121	9.31 ± 6.42		62.02 ± 109.19	
Gender			0.58		0.69
• Man	75	9.39 ± 0.70		68.11 ± 13.96	
•Women	46	9.17 ± 1.03		54.72 ± 12.86	
Profession			0.54		0.48
• Student	4	6.75 ± 1.43		17.00 ± 4.91	
• Housewife	38	8.61 ± 0.74		49.89 ± 10.96	
• Retired	43	9.14 ± 0.67		53.84 ± 9.05	
• Officer	12	8.92 ± 2.27		61.67 ± 40.23	
• Worker	18	9.06 ± 1.43		70.39 ± 26.63	
• Tradesman	6	18.17 ± 7.04		223.17± 130.26	
Body massindex			0.40		0.14
• Underweight	1	---		---	
• Normal weight	35	9.24 ± 1.30		41.79 ± 13.67	
• Overweight	49	8.41 ± 0.66		44.54 ± 10.28	
• Obesity	32	11.64 ± 1.41		130.11 ± 31.53	
• Severelyobese	4	6.75 ± 0.75		9.24 ± 1.30	
Diabetesmellitus			0.06		0.08
• Present	25	11.32 ± 6.9		93.04 ± 12.29	
• Absent	96	8.78 ± 6.2		60.93 ± 11.79	
Hypertension			0.44		0.34
•Present	30	9.93± 1.20		78.83 ± 25.84	
•Absent	91	9.10± 0.66		57.80± 10.12	

* CSD was calculated by measuring the longest side of stones (in mm) for patients with a single stone and by adding together the sizes of longest stone side in patients with more than one stone.

** SA was calculated from the formula stone diameter × width × Ω × 1/4 (in mm^2^).

*** Statistically significant at p < 0.05.

## DISCUSSION

The incidence and prevalence of kidney stones are increasing in recent years. Research into kidney stone disease continues to gain importance due to the potential for the disease to cause serious morbidity and high treatment costs. Major risk factors for stone formation include age, gender, ethnicity, family history, history of stones, hypercalciuria, hyperoxaluria, hypocitraturia and urinary pH disorders.^12^,^13^ It is thought that inappropriate dietary habits, increase in prevalence of overweight people and changes in lifestyle are responsible for the increase in kidney stone prevalence in the last 2-3 decades.^2^,^9^ Epidemiological studies have shown that DM, metabolic syndrome and obesity increase the risk of kidney stone disease.^7^,^14^

Metabolic syndrome is an important health problem affecting 20-30% of the general population.^8^ Parameters evaluated for metabolic syndrome are blood pressure, blood lipid profile (triglyceride and HDL) and abdominal obesity together with dysfunctional glucose tolerance or insulin resistance. MS is known to cause more uric acid stone formation, but studies in recent years indicate it increases the risk for calcium stones.^8^,^15^ The relationship of components of MS, diabetes, hypertension and obesity, to formation of kidney stones has been shown by previous studies.^7^,^12^,^14^,^16^-^19^ The insufficient response of peripheral tissues to insulin in circulation, known as insulin resistance, was held responsible for the pathophysiology of stone formation in these diseases. Analyses completed found a significant statistical relationship between presence of stone and hypertension, diabetes and BMI. However it was found that these diseases did not increase stone burden.

The relationship between hypertension and kidney stone was first described by Morgagni in the year 1761.^17^ Madore et al. in a prospective study found that the risk of developing HT increased after kidney stones.^20^ In a study by Capuccio et al. at the end of an 8 year follow-up period they found the risk of development of kidney stones was high in HT patients.^17^ Potential mechanisms are thought to be variable sodium involvement related to renal tubular defect causing calcium leak and following this, the development of secondary parathyroid activation. In our study when the presence of HT in the group with stones found on ultrasound is compared to the rate of HT in the group without stones, a statistically significant difference was observed (p=0.007). In a study with broad participation comparing the characteristics of metabolic syndrome with severity of kidney stone disease, it was found that HT was related to the severity of stone disease (recurrent and/or multiple stones) independent of other variables.^21^ However, in our study there was no significant relationship found between CSD and SA, which can indicate severity of stone disease, and HT. As a result intervention for early diagnosis and the treatment received by patients under close observation due to HT may have led to them not being included in our study.

Diabetes mellitus is a common disease that may cause a variety of urinary system disorders such as nephropathy, infection and motility disorders. Current studies have shown a positive correlation between diabetes and kidney stones.^14^,^16^,^19^ In conclusion increased urine acidification, hypocitraturia, hyperoxaluria, hyperphosphaturia and hypercalciuria are among the pathophysiological mechanisms responsible for formation of stones in diabetes.^7^,^14^,^16^

In prospective research involving 3 large cohort studies with more than 200,000 participants, Taylor et al. evaluated the relationship between diabetes and kidney stones. Variables were evaluated with multi-variate analysis and diabetes was found to be related to stone formation independent of BMI, age, thiazide use and diet.^16^ The result of this study is that in advancing years people with a history of kidney stones have increased possibility of diabetes. A study by Meydan et al. found no difference in stone incidence according to radiological evaluation (DUSG, US and/or IVP) between those with and without diabetes.^19^ They indicated that the close medical follow-up of the diabetic patient group may lead to early diagnosis and treatment of kidney stones. However, this study did not separately evaluate those positive for stone history and those with stones found on radiological evaluation which limits the study. Due to the difficulty of evaluating stone size and the subjective data of previous stone size, we did not include patients with stone history in our study. In accordance with the literature in our data while 20.6% of patients with kidney stone identified by ultrasound had DM, only 10.8% of patients without stones had DM (p=0.004). According to logistic regression analysis, if the person has diabetes, estimated relative risk is increased 2.06 times more. In a cross-sectional study of adults participating in NHANES from 2007 to 2010 the relationship between severity of DM and kidney stones was researched.^14^ Even when variables such as age, gender, ethnicity and BMI were equilibrated, from the point of view of HbA1c, fasting blood sugar and fasting blood insulin, there was no correlation found between severity of diabetes and presence of stones. In our study we evaluated the stone burden of patients with and without diabetes to show severity of stone disease. While the average CSD in patients with DM was 11.32 ± 6.9 mm (n=25), the average CSD for patients without DM was calculated as 8.78 ± 6.2 mm (n=96) (p=0.062). The SA of diabetic patients was calculated as 93.04 ± 122.9 mm^2^, while for non-diabetic patients it was 60.93 ± 117.90 mm^2^ (p=0.084).

Another study recently researched intensively for relationship to stone formation is BMI. In previous studies it has been stated that higher body mass index (BMI) is observed to be associated with increased prevalence of cardiovascular disease in the general population.^22^ But, first Taylor et al. in a large prospective cohort study found the incidence and prevalence of kidney stone was directly related to weight and BMI.^23^ In obese patients as a result of hyperinsulinemia and insulin resistance related to visceral obesity, dysfunction in ammonium excretion and urine acidification in the proximal tubule caused low urinary pH. They showed a negative relationship between urinary pH and body size. Eisner et al. in a retrospective study of 880 patients found that increased BMI was related to a variety of risk factors for kidney stone disease.^18^ In our study BMI values for patients with and without stones were 27.2 ± 4.93 kg/m^2^ and 25.29 ± 4.12 kg/m^2^, respectively (p<0.001). A study by Lee et al. determined that uric acid stones are more frequently seen in obese patients. Additionally the obesity of patients with stones formed for the first time was revealed as a strong predictor of stone recurrence.^12^ In our study there was no significant relationship found between stone burden and BMI.

Studies in recent years have clarified the role of HT, DM and increased BMI among the risk factors for stone formation. They cause stone formation by changing the composition of urine by different mechanisms. In our study in accordance with the literature, significant relationships between HT, DM, BMI and presence of stone were found. The relationship between HT and obesity with severity of stone disease (multiple and/or recurrent stone disease) has been shown by previous studies.^12^,^20^ Again the correlation between severity of DM and presence of stone was proven.^13^

Looking at previous data to show a similar relationship between DM, HT and BMI and severity of stone disease, there was no statistically significant difference found when stone burden was compared. This may be due to intervention to diagnose this patient group due to current diseases before stones grow as patients with stone history were excluded from our study.

There are some natural limitations to our study. The first is the retrospective nature of the study. The second is that the height, weight and blood pressure of patients were self-reported. Lastly patients included in the study visited the urology clinic with flank pain, so the incidence may be different compared to a community-based population. Therefore, larger prospective randomized studies are required to confirm these findings.
